# Characterization of a PCB Based Pressure Sensor and Its Joining Methods for the Metal Membrane

**DOI:** 10.3390/s21165557

**Published:** 2021-08-18

**Authors:** Adrian Schwenck, Tobias Grözinger, Thomas Günther, Axel Schumacher, Dietmar Schuhmacher, Kai Werum, André Zimmermann

**Affiliations:** 1 Hahn-Schickard, Allmandring 9b, 70569 Stuttgart, Germany; Tobias.Groezinger@Hahn-Schickard.de (T.G.); Thomas.Guenther@ifm.uni-stuttgart.de (T.G.); Kai.Werum@Hahn-Schickard.de (K.W.); Andre.Zimmermann@ifm.uni-stuttgart.de (A.Z.); 2 Institute for Micro Integration (IFM), University of Stuttgart, Allmandring 9b, 70569 Stuttgart, Germany; 3 Hahn-Schickard, Wilhelm-Schickard-Str. 10, 78052 Villingen-Schwenningen, Germany; Axel.Schumacher@Hahn-Schickard.de (A.S.); Dietmar.Schuhmacher@Hahn-Schickard.de (D.S.)

**Keywords:** pressure sensor, capacitive sensor, media resistant, SMD process, PCB based sensor, reactive joining, transient liquid phase bonding, sintering, welding

## Abstract

Essential quality features of pressure sensors are, among other accuracy-related factors, measurement range, operating temperature, and long-term stability. In this work, these features are optimized for a capacitive pressure sensor with a measurement range of 10 bars. The sensor consists of a metal membrane, which is connected to a PCB and a digital capacitive readout. To optimize the performance, different methods for the joining process are studied. Transient liquid phase bonding (TLP bonding), reactive joining, silver sintering, and electric resistance welding are compared by measurements of the characteristic curves and long-term measurements at maximum pressure. A scanning electron microscope (SEM) with energy-dispersive X-ray spectroscopy (EDX) analysis was used to examine the quality of the joints. The evaluation of the characteristic curves shows the smallest measurement errors for TLP bonding and sintering. For welding and sintering, no statistically significant long-term drift was measured. In terms of equipment costs, reactive joining and sintering are most favorable. With low material costs and short process times, electric resistance welding offers ideal conditions for mass production.

## 1. Introduction

Pressure sensors are among the top-selling microsystem components and sensors [1] due to their advantages of small sizes, high resolutions, and low costs [2]. Application areas where they are widely used are automotive industry, medical instrumentation, aerospace, automation technology, and military [2]. Specific examples of applications in aerospace are pressure sensing in aircraft turbines and space propulsion combustion chambers [3], altimetry, and speed measurement with pitot velocimeters [4]. Automotive applications include pressure monitoring in tires and pneumatic truck brake systems, crash detection, and monitoring of exhaust emission control devices of internal combustion engines. Typical applications for absolute pressure sensors in consumer electronics are barometric altimetry in indoor navigation and fitness tracking, and environmental monitoring [5]. In automation technology, hydrostatic level measurement and monitoring fluid flow is a common application for media-resistant pressure sensors [6].

Important sensor technologies are metal thin films, ceramic thick films, and silicon microelectromechanical systems (MEMS) [7]. Table 1 compares the different sensor technologies. MEMS pressure sensors can be built with a hermetically sealed reference vacuum to measure absolute pressure [8].

The most common measurement principles for pressure sensors employ capacitive and piezoresistive effects [9]. To measure dynamic changes, piezoelectric sensors can be used, but they are not sensitive for static pressure [7]. Recent developments for highly accurate sensors are based on resonant detection mechanisms [10,11].

Important technologies for pressure sensors are metal thin film, ceramic thick film and MEMS. While metal thin film pressure sensors show excellent performance for very high pressure, shock and vibration resistance, as well as long term stability, they are very limited in measuring absolute pressure and low pressure. MEMS pressure sensors in contrast are able to perform measurements of absolute pressure and very low pressure, but fail when it comes to very high pressure. Ceramic pressure sensors fulfill all criteria but cannot be employed for very low as well as very high pressure ranges [7].

The common disadvantage of all the prevailing sensor technologies is the necessity for costly investments in production equipment, which presents a major difficulty for small and medium enterprises (SMEs) to enter the market. Therefore, other concepts are necessary for SMEs to benefit from the large projected growth in the pressure sensor market [1] or to increase the vertical integration of manufacturing by integrating sensor technology. The capacitive pressure sensor in this work offers a solution for SMEs to increase vertical integration and to reuse already available surface-mount device (SMD) assembly machines, since only low-cost production technologies are required.

MEMS pressure sensors represent the second-highest market volume among all MEMS [1], but they are not suitable for all applications. Most applications require complex and, thus, expensive housings, e.g., for the separation of the process medium with a stainless steel membrane and a transmission fluid [7,12] or the use of a silicone gel [13] to protect the MEMS device from environmental influences. In such cases, the manufacturing cost of the MEMS sensor only represents a minor amount of the total sensor system costs. In order to lower the system costs and to enable SMEs to produce the pressure sensors in house by simplifying the construction and the production, a new capacitive pressure sensor with a metal membrane on printed circuit board (PCB) is proposed.

**Figure 1:**
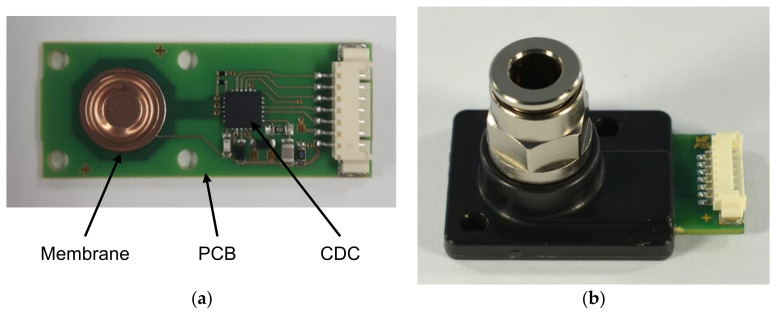
Images of the capacitive pressure sensor (**a**) without housing; (**b**) with housing for relative pressure measurement.

**Figure 2:**
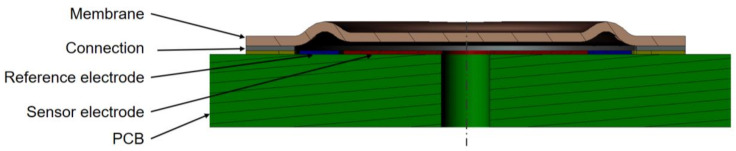
Functional principle of the pressure sensor (sketch, cross section).

The presented pressure sensor consists of a PCB, a metal membrane, and a capacitive readout circuit (Figure 1). The membrane is connected to the PCB by soldering or other methods, which are investigated in this work (Figure 2). The shape of the membrane forms a narrow gap to the electrode located on the PCB. The pressure difference from top to bottom of the membrane affects the distance between the membrane and the electrode.

**Figure 3:**
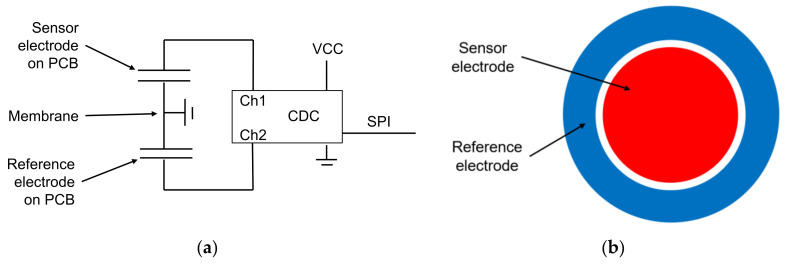
Description of the sensor element: (**a**) electrical diagram of the sensor; (**b**) electrode layout of the PCB (schematic).

The displacement is detected by measuring the capacitance between the membrane and the electrode with a capacitance to digital converter (CDC) PCap01 (SioSense, formerly acam messelectronic). The electrical diagram is shown Figure 3.

Two electrodes are located beneath the membrane on the PCB. The inner one is located under the flexible part of the membrane and is used for the distance measurement. The outer one is circular arc shaped and located under the waved and stiff area of the membrane and acts as a reference, compensating the influence of temperature and humidity of the air inside the sensor cell. The output signal is the difference between the sensor and the reference capacitance.

The CDC (Table 1) is based on the measurement of the discharge time of an RC circuit [14]. When using a fixed discharge resistor, the capacitance can be determined. By recording the cycle time of logic gates for time quantization, the CDC achieves a temporal resolution in the picosecond range [14].

**Table 1:** PCap01 specifications; Data extracted from [14].

Quantity	Value
Measurement range	Variable ^1^
Measurement mode	Grounded or floating
Number of channels	Up to 8
Output data rate	Up to 500 kHz+
RMS noise (output data rate = 10 Hz, grounded)	11 aF
Interface	SPI
Temperature range	−40 … +125 °C
Temperature sensor	Integrated or external PT1000

^1^ dependent on discharge resistor.

Depending on the material of the membrane, measurements in aggressive media are possible. In this work, membranes made of 1.4435 stainless steel and copper–tin alloy CuSn8 are employed. The printed circuit boards (PCBs) are metallized with 35 µm copper (Cu) and electroless nickel immersion gold (ENIG) coating. Dependent on the type of the housing, differential or relative pressure can be measured. To measure absolute pressure, a hermetically sealed reference vacuum is necessary. Since printed circuit boards are not hermetically sealed, other base materials are required. Thus, this work focuses on a sensor for relative pressure (Figure 1b).

Previous work on this pressure sensor principle with SAC305 lead-free soldered membranes shows good results in the pressure range from 0 to 1 bar [15]. However, with exposure to higher static pressure, drift effects occur within hours. Furthermore, various industrial applications require a higher range. An analysis showed highest demand for a frequent measuring range up to 10 bars due to the fact that pneumatic applications in particular take place in this pressure range.

**Figure 4:**
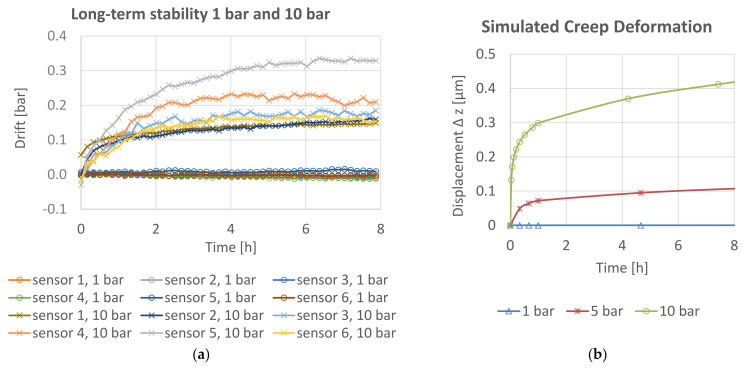
(**a**) Measured long-term drift of sensors with SAC305 soldered membrane at 1 bar and at 10 bars at room temperature; (**b**) simulated creep deformation for SAC305 solder at 20 °C.

For SAC305 lead-free solder alloy, the homologous temperature at 25° is 0.61, and thus creep deformation is a dominant factor. This corresponds to the measurements in Figure 4a and to the FEM simulation in Figure 4b. To overcome that issue, other methods to join the membrane on the PCB are considered in this work. Namely, these are silver sintering, transient liquid phase bonding (TLP bonding), reactive joining, and welding.

To simulate the mechanical behavior of the pressure sensor, especially drift effects in the interconnection between the membrane and the PCB, a finite element method (FEM) simulation with Ansys^®^ Workbench was conducted. Garofalo’s equation for transient creep deformation (Equation (S1), Tables S1 and S2, Figures S1–S3) and creep deformation curves from [16] were used to describe the long-term material behavior [17]. The simulation result (Figure 4b) shows a qualitatively good correlation with the measured curves.

Time-dependent deformation in the membrane can be excluded, as measurements of the viscoelastic deformation of the membrane material CuSn8 (Figure 4b) showed no long-term drift within the relevant level of stress.

**Theorem** **1.** *The drift effect of the sensor signal at high static pressure is related to creep deformation in the solder joint between the membrane and the PCB. The sensor signal can be optimized by changing the joining process.*

## 2. Materials and Methods

### 2.1. Joining Processes for the Membrane

Different joining processes can be used for electronic packaging. Important requirements for the joining process for the membrane and the PCB are little or no tendency for creep deformation, low material and process costs, mechanical stability, and process temperatures that do not damage the PCB.

SAC solder was used in previous work [15]. The most relevant drawback is the low melting temperature and the resulting tendency for creep deformation.

A high-temperature reflow solder process in a convection or vapor-phase oven is not suitable for the pressure sensor, as the process temperature exceeds the maximum temperature permissible for the PCB. High temperature solder can only be used with local heating, e.g., with reactive joining. Another possibility is to change the base material, e.g., employing ceramics. However, as the sensor in this work focuses on the low-cost market for media resistant pressure sensors, ceramics are not further considered.

The process temperature of silver sintering and TLP bondig is 250–280 °C and 200–300 °C, respectively [18]. That is within the temperature range of the materials used in this work. The temperature stability of both joining methods is well above the process temperature. This indicates a good suitability for the application.

#### 2.1.1. TLP Bonding

**Figure 5:**
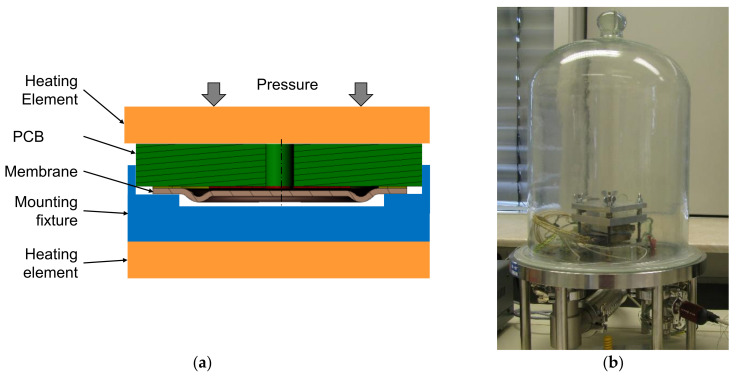
TLP bonding process: (**a**) cross-section sketch; (**b**) apparatus for TLP bonding in vacuum used in this work.

TLP bonding is based on diffusion processes and the formation of intermetallic phases [19]. Pressure, temperature, and vacuum is needed during the whole process (Figure 5). The main advantage is that the melting point of the resulting connecting alloy is significantly higher than the process temperature of the bonding process. It is used, for example, in microelectronic packaging for high-temperature applications [20]. TLP bonding is described in detail in [18,19,20,21].

**Figure 6:**
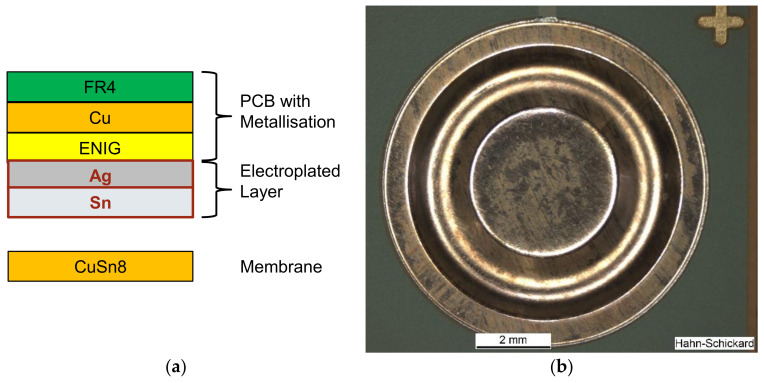
TLP bonding: (**a**) cross-section sketch of the layer structure; (**b**) top view of a TLP-bonded membrane.

In this work, the layer structure, which is shown in Figure 6, was used. The PCB metallization is covered with an additional layer of 12 μm silver (Ag) and 6 μm tin (Sn). This layer is connected with the CuSn8 membrane during the TLP process by the formation of intermetallic phases. The possible resulting alloys are Cu_6_Sn_5_, Cu_3_Sn, and Ag_3_Sn. The combination of Ag–Sn was chosen as the investigations of [22] revealed a reduction of pores in the TLP bond and improvement of the mechanical properties of the TLP bond compared to pure Ag or pure Sn. The process parameters used in this work were 250 °C temperature, 30 min process time, 13.8 MPa pressure, and 2.2 mbar vacuum.

#### 2.1.2. Reactive Joining

**Figure 7:**
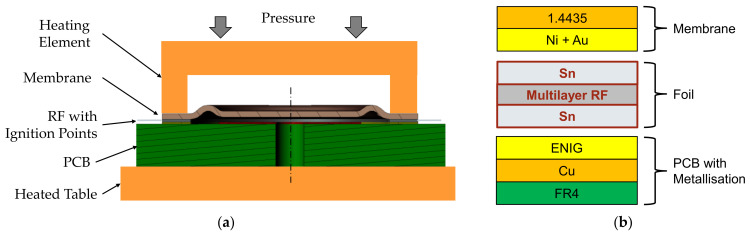
Reactive joining process: (**a**) cross-section sketch of the equipment; (**b**) cross-section sketch of the layer structure.

Reactive joining uses an exothermal reaction to generate the heat required for soldering. As the heat is generated only locally, heat-sensitive materials can be connected. For soldering, a reactive foil (RF) is stacked between two layers of solder (Figure 7b). The reactive foil is a multilayer metal. Different material combinations with different amounts of thermal energy are possible. Reactive bonding can be adapted to a wide range of soldering materials. The activation energy to start the reaction is commonly provided by an electric spark (Figure 7a). Joining of dissimilar materials, e.g., borosilicate glass, silicon, aluminum oxide, and metals, is possible. Typical applications are connecting sensor chips onto larger components and thermal management in power electronics [23].

**Figure 8:**
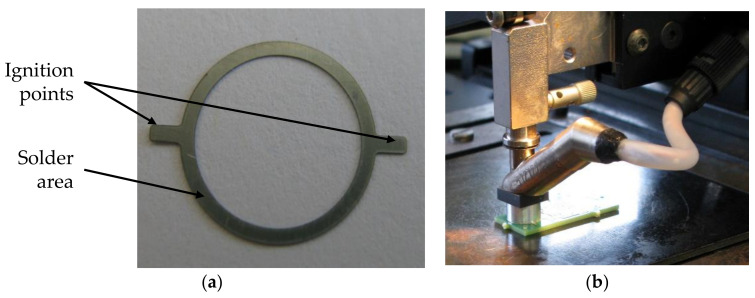
Reactive joining: (**a**) RF preform; (**b**) flip chip bonder with heated placement head used for reactive joining in this work.

The layer structure used in this work is shown in Figure 7b. Here, 1.4435 stainless-steel membranes with sputtered nickel–gold (Ni+Au) coating and nickel–aluminum (Ni–Al) reactive foils (RF) coated with tin (Sn) solder were used. To match the form of the solder joint and to attach the ignition points, the foil was structured by laser cutting. That preform (Figure 8a) was positioned together with the membrane onto the metallization of the PCB. Figure 8b shows the setup for reactive joining used in this work. A flip chip bonder (FINEPLACER^®^, Finetech GmbH, Berlin, Germany) was used for positioning and application of pressure during the joining process.

#### 2.1.3. Silver Sintering

Silver (Ag) sintering is often used as an interconnection technique when high-temperature resistance of 200 °C and more is required, and it presents a possible replacement for high-melting solder. Typical applications are found in power electronic components of hybrid and electric cars, high-speed trains, aircraft and aviation, and deep-well oil and gas extraction [24].

Sintering is based on diffusion and takes place below the melting temperature of the materials. Micro- or nanosized particles are needed, as the process is driven by the reduction of the total surface energy. The substrate and the sintered Ag are connected with a diffusion intermetallic phase. Usually, high pressure of up to 40 MPa is applied to the materials to assist the bonding during the process when microsized particles are used. Investment costs and manufacturing throughput can be optimized by using pressureless sintering with nanosized particles [24].

In this work, CuSn8 membranes were sintered with the nanosized Ag sintering paste NJ-ONE (Nano-Join) to the PCB. The membranes were coated with CuTi/Au by sputtering to optimize the adhesive strength. The silver sintering paste was stencil printed on the PCBs, then the membrane was assembled with a pick-and-place machine. After the initial placement, no pressure was applied during the sintering process. The sintering process was conducted in a conventional convection oven at 250 °C for 60 min.

#### 2.1.4. Electric Resistance Welding

Welding is based on melting and merging of two metal parts. Resistance spot welding uses an electric current to generate heat at the contact point of the materials. It is widely used in automotive and aerospace industry, in the production of white goods, and in steel construction. It is particularly suitable for high-volume production due to excellent automation capability and low cycle time resulting in low operating cost [25].

**Figure 9:**
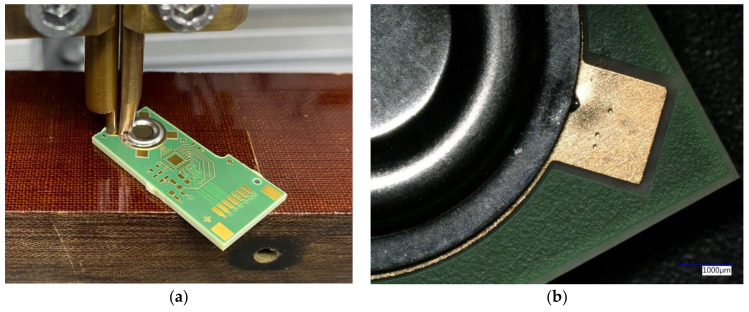
Electric resistance welding: (**a**) welding electrodes; (**b**) single-step welding spot; current = 500 A; pulse duration = 2.5 ms.

In this work, a welding machine with modular pneumatic weld heads from AMADA WELD TECH was used. In order to weld the membrane on the metallization on the PCB without damaging the PCB, the process parameters pressure, current, and time were optimized. Electrodes made of copper chromium zirconium alloy were used to weld 1.4435 stainless steel membranes on a PCB with 35 µm copper and ENIG surface metallization. Four welding spots, each with one electrode on the metallization of the PCB and one electrode on the membrane (step welding), are evenly distributed around the circumference of the membrane (Figure 9).

#### 2.1.5. Comparison of the Joining Processes

This chapter compares the necessary equipment and materials for the joining processes. The influence on the sensor performance is described in Section 3. Table 2 compares the processes on the machines used in this work. The use of equipment for series production results in different values. For TLP bonding in particular, the process time is strongly dependent on the used equipment. Active cooling of the heating plates significantly reduces the process time, while the bonding time without heating and cooling takes a few minutes.

Equipment costs for TLP bonding are high, as the process requires pressure and vacuum, and the sensors must remain in the device for the entire duration. In contrast, sintering, which is carried out in this work without external pressure to reduce machinery costs, can be performed in a batch in a convection oven. The application of the sintering paste by squeegeeing is also inexpensive. Reactive joining requires very low process energy, due to the rapid process, and can be performed with low-cost equipment; pressure is needed for only a few seconds. Since controlled high peaks of energy within a short period of time are required, equipment cost for an electric resistance welding machine is high compared to the other processes.

The operational costs of electric resistance welding, on the other hand, are the lowest, as no additional material is needed and only the electrode wear needs to be considered. For silver sintering, a relatively costly sintering paste is necessary. The RF nanoscale multilayer foil with solder coating is costly as well, and has to be laser trimmed to the exact shape of the connection. For TLP bonding, the PCBs must be coated by electroplating, which increases the costs per unit.

### 2.2. Characterization

The capacitive measurement principle is sensitive to temperature and humidity changes in the sensor element and the CDC. To overcome that issue, the sensor cell comprises a measurement and a reference electrode. The difference between the two capacities is the output signal of the sensor.

To further minimize environmental influences, the measurements were performed in a climate-controlled laboratory. The temperature and the humidity of the laboratory were recorded with a data logger. The sensors also have an internal temperature sensor. Possible temperature or humidity influences can be seen in the characteristic curve. In this case, the measured value at the start and at the end of the test would differ significantly. This was not observed in the measurements, which were included in the evaluation.

#### 2.2.1. Determination of Accuracy-Related Factors

**Figure 10:**
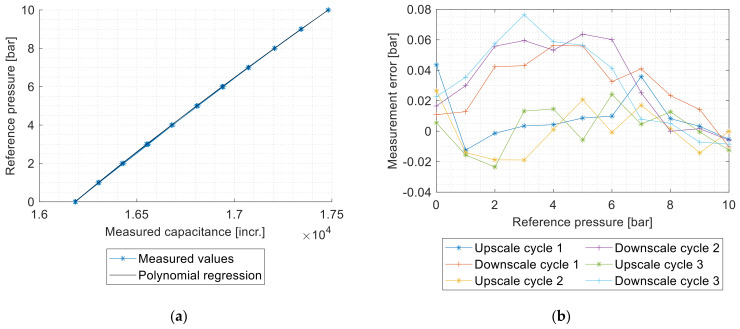
Measurement result. (**a**) Characteristic curve with polynomial regression: the measured values were fitted with a second-order polynomial approximation to calibrate the values and to obtain the measured pressure values. (**b**) Error curve: the measured values for upscale and downscale cycles are shown separately, so that a possible hysteresis can be identified.

The international standard IEC61298-2 [26] describes the methods to evaluate the performance of sensors. The characteristic curves were recorded with the pressure controller described in Section 2.2.4. Three measurement cycles with 11 test points were conducted according to the standard. The capacitance values were calibrated with a second-order polynomial approximation with the method of least squares to calculate the pressure. The difference between the reference pressure and the pressure measured by the sensor results in the measurement error (Figure 10).

**Figure 11:**
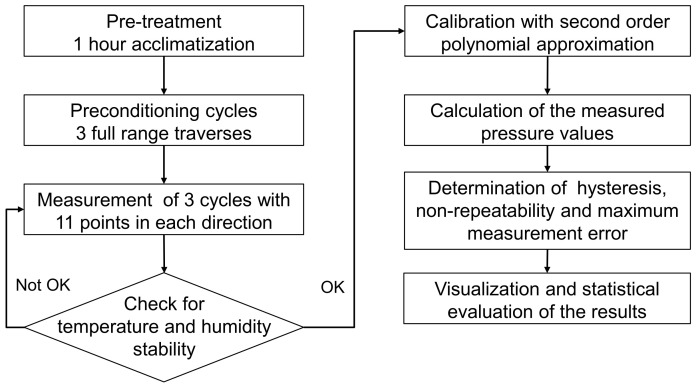
Flowchart of the measurement procedure for the characteristic curves.

In this work, the accuracy related factors—maximum measured error, nonrepeatability, and hysteresis—were determined, based on the error curve and according to the standard. All these values are expressed as percentage of the full-scale output (% FSO). The measurement and evaluation procedure is shown in Figure 11.

Nonrepeatability is the difference between the extreme values obtained by a number of consecutive measurements for the same value of input approaching from the same direction. Hysteresis is not included in the nonrepeatability. The difference between consecutive up- and downscale outputs at the same test point is called the hysteresis. The maximum measured error is the greatest positive or negative measured error [26].

#### 2.2.2. Determination of Drift Effects

**Figure 12:**
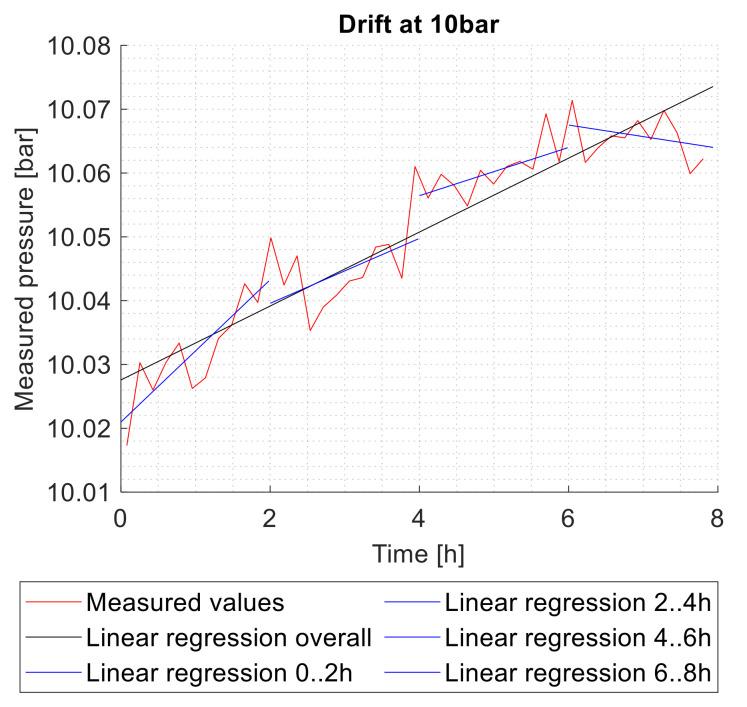
Drift at 10 bars reference pressure with linear regression lines.

To determine drift effects, maximum pressure was applied to the sensors for 8 h. During that time, the measured pressure was recorded. The slope of the linear regression lines, which are determined by the method of least squares, was used to evaluate and to compare the drift. To show the chronological trend of the drift, the slope was determined for time intervals of two hours and additionally for the entire measurement period (Figure 12). The drift is specified in % FSO/h.

#### 2.2.3. T-Test

The *t*-test is a statistical hypothesis test. It can be used to compare one set of values with a reference value, µ_0,_ with the one-sample *t*-test, or to compare two sets of data with each other with the two-sample *t*-test [27].

In this work, the one-sample *t*-test was used to determine if the measured long-term drift exhibits a statistically significant difference from µ_0_ = 0. The independent two-sample *t*-test with unequal variances and unequal sample sizes was used to compare the results of the measurements of the characteristic curves with different joining processes. A normal distribution of the measured values is assumed. The *p*-values were calculated with a statistics program, e.g., Excel. An effect is significant if the *p*-value is smaller than 0.05, which approximately corresponds to a 2σ confidence interval [27].

#### 2.2.4. Measurement Equipment

A pneumatic precision pressure controller (CPC6000, WIKA Alexander Wiegand SE & Co. KG, Klingenberg, Germany) with an integrated reference sensor with 20 bar measurement range was used to perform the measurements. The control stability of the pressure controller is 0.003% FS and the accuracy is 0.01% FS [28]. Therefore, the measuring device is more than one order of magnitude more accurate than the sensors to be measured. The manufacturer recently calibrated the pressure controller according to IEC 61298 and issued a certificate of acceptance according to EN 10204-3.1.

## 3. Results

### 3.1. Characteristic Curve

**Figure 13:**
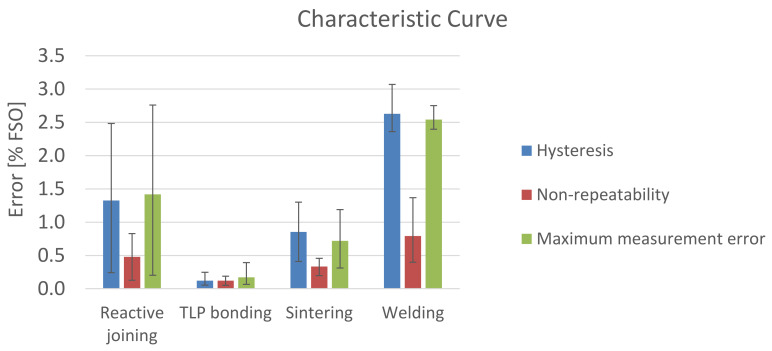
Results of the measurement of the characteristic curve.

The characteristic curve was measured and the accuracy related factors, hysteresis, nonrepeatability, and maximum measured error were evaluated according to the international standard IEC 61298-2 [26]. The results are shown in Figure 13.

The measurement results for reactive joining fluctuate the most. The standard deviation (SD) of the maximum measurement error is 0.885% FSO for reactive joining, 0.101% FSO for TLP bonding, 0.303% FSO for Ag sintering, and 0.125% FSO for welding. Yet the average (mean) maximum measurement error of 1.417% FSO is below welding, with 2.541% FSO. The difference is statistically significant (t(8) = −3.75; *p* = 0.006).

Sensors with membranes which were connected by TLP bonding with the PCB show the best characteristic curve. Compared to sintering (mean = 0.719; SD = 0.092), TLP bonding (mean = 0.170; SD = 0.010) is significantly better (t(10) = −5.18; *p* = 0.0004).

Hysteresis is the main factor influencing the maximum measurement error.

### 3.2. Drift

**Figure 14:**
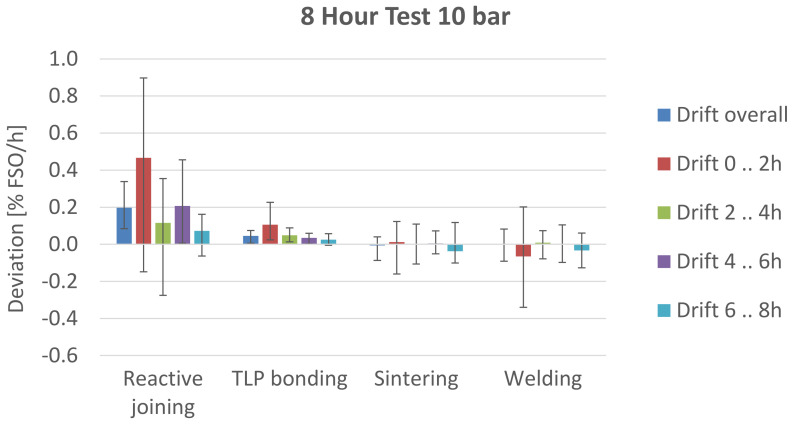
Results of drift test.

The results of the long-term drift test are shown in Figure 14.

The one-sample *t*-test shows a significant difference between the overall drift and µ_0_ = 0 for reactive joining (mean = 0.20; SD = 0.10; t(8) = 5.65; *p* = 0.0005) and TLP bonding (mean = 0.05, SD = 0.02; t(8) = 5.82; *p* = 0.004). For sintering (mean = −0.01; SD = 0.04; t(8) = −0.461; *p* > 0.6) and welding (mean = 0.00; SD = 0.08; t(5) = −0.009; *p* > 0.99), no significant overall long-term drift was measured. If a drift is measured, most of the deviation occurs in the first 2 h of the measurement. This corresponds to the expected behavior for creep deformation.

### 3.3. Overpressure

**Figure 15:**
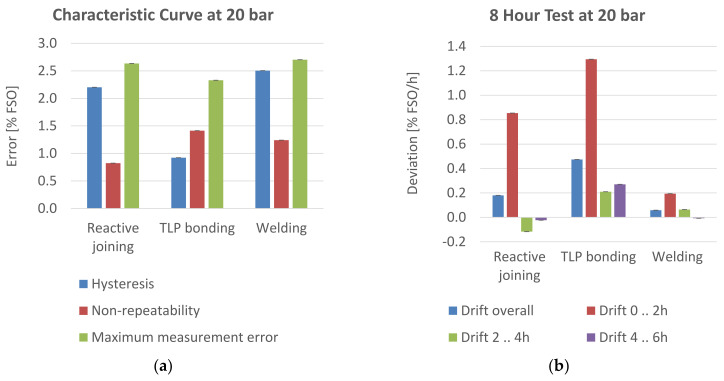
Characterization at 20 bar overpressure; (**a**) characteristic curve; (**b**) drift test.

To investigate the behavior of the sensors at overpressure, one sample each was characterized at 20 bars. The sensor with the joining method sintering failed in the test. The membrane was no longer attached to the PCB after the opening of the housing after the test. The measurement results of the other sensors are shown in Figure 15.

The sensor with the welded membrane performed best, regarding the drift test. The characteristic curve does not differ significantly from the one at 10 bars. With regard to hysteresis, the sensor with the TLP-bonded membrane showed the smallest deviations from the nominal value.

### 3.4. Joining Process

A scanning electron microscope (SEM) with energy-dispersive X-ray spectroscopy (EDX) (JEOL GmbH JSM-6490LV) was used to obtain images of the joints and to analyze the elemental distribution in the cross section of the sensor samples. The aim was to investigate the joint for pores, cracks, or voids, for diffusion processes and for the formation of intermetallic phases. Despite the far higher resolution of the SEM image, the spatial resolution of the EDX is limited to 2 µm. Blurred transitions at obvious interconnections in the element analysis and difficulties in the identification of intermetallic phases are results of the limited EDX resolution.

#### 3.4.1. TLP Bonding

**Figure 16:**
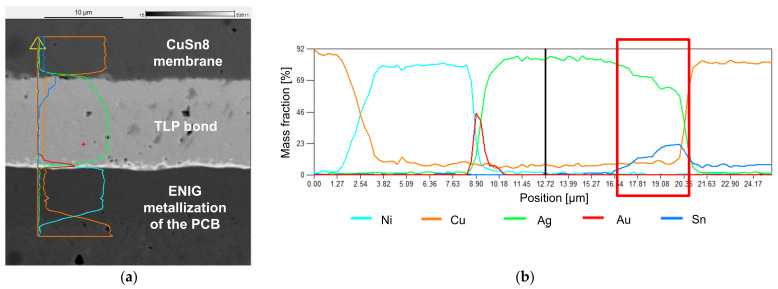
SEM-EDX image with elemental analysis of the TLP bond: (**a**) image with indication of the position of the EDX line scan; (**b**) EDX elemental analysis of the bond (line scan).

The SEM image (Figure 16) of the TLP bond shows a good connection, with few pores or voids and without cracks. In the highlighted area at the interconnect to the membrane, silver, copper, and tin are present. The maximum concentration of tin is in the interconnect between the TLP bond and the membrane. This indicates a formation of intermetallic phases between Ag, Sn, and Cu. The exact composition of the alloys cannot be concluded from the analysis.

#### 3.4.2. Reactive Joining

**Figure 17:**
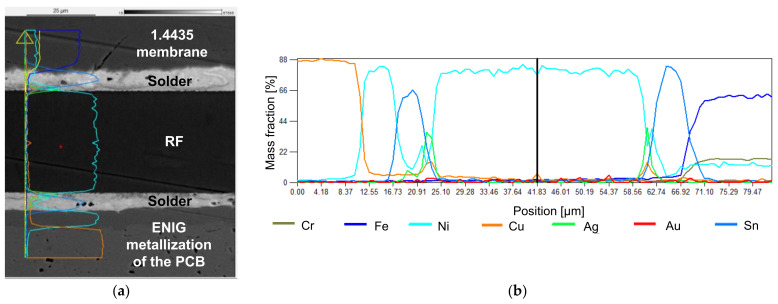
SEM-EDX image with elemental analysis of reactive joining: (**a**) image with indication of the position of the EDX line scan; (**b**) EDX elemental analysis of the bond (line scan).

The thin solder layer of a few µm can be seen in Figure 17. The thinner layer should reduce creep deformation compared to conventional SAC soldering. In the Sn solder, a few pores are visible. At the interconnections between the materials, no defects can be spotted. Due to the limited resolution of the EDX, the intermetallic phases cannot be clearly identified.

#### 3.4.3. Sintering

**Figure 18:**
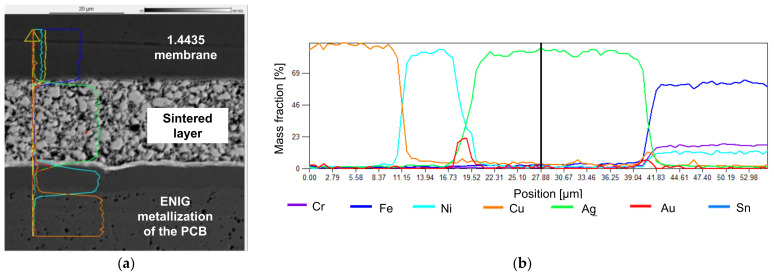
SEM-EDX image with elemental analysis of sintering: (**a**) image with indication of the position of the EDX line scan; (**b**) EDX elemental analysis of the bond (line scan).

The typical pores for sintered materials are clearly visible in the SEM image (Figure 18). Both interconnections of the sintered material are without cracks or bigger pores.

#### 3.4.4. Welding

**Figure 19:**
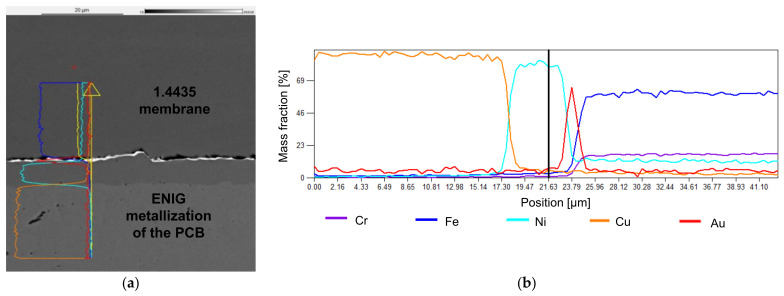
SEM-EDX image with elemental analysis of welding: (**a**) image with indication of the position of the EDX line scan; (**b**) EDX elemental analysis of the bond (line scan).

The SEM image shows the welding connection between the membrane and the PCB (Figure 19). Many unconnected areas are visible at the interconnect between the membrane and the metallization. Further optimization of the welding parameters could improve the mechanical connection.

## 4. Discussion

Creep deformation is a temperature-dependent effect. The tendency of a material to undergo creep deformation can be calculated with the homologous temperature T_H_ [29] (p. 393).

T_H_ = T/T_M_ (1)

T is the operating temperature, in Kelvin, and T_M_ is the melting temperature, in Kelvin, of the material. The minimum temperature at which creep deformation can occur is T_H_ = 0.3–0.4. The maximum operating temperature of crystalline metals for mechanically highly stressed applications is around T_H_ = 0.5 [29] (p. 396). Creep deformation is a dominant factor of the material properties [30] above T_H_ = 0.6 [30]. In Table 3, the homologous temperature for the materials used in this work is calculated for operating temperatures of 25 °C and 85 °C.

The membranes used in this work are fabricated by deep drawing. The used materials are 1.4435 and CuSn8. With T_H_ < 0.5, the membranes are most likely not the reason for the measured creep deformation.

Pure tin was used as solder material in the reactive joining process in this work. The homologous temperature is only a little lower than the one of SAC305 solder, hence creep deformation is expected to be an issue, too.

Another possibility to connect the membrane on the PCB is silver sintering. For the calculation of the homologous temperature, the melting temperature of bulk silver T_M_ = 1235 K [32] (p. 113) is not relevant, as the particle size of a material affects the melting temperature [35]. For silver sintering, the micro/nano size remelting temperature is >900 °C [18]. The resulting homologous temperature is below 0.5, and creep deformation is not expected to be dominant.

The main advantage of TLP bonding is the resulting melting point of the connecting phase, which is above the bonding temperature [21]. The expected resulting alloys in this work are Cu6Sn5, Cu3Sn, AG85Sn15, and Ag3Sn. The melting temperatures can be obtained from the phase diagrams for Cu–Sn and Ag–Sn. Of these alloys, Cu6Sn5 has the lowest melting temperature at ≈610 °C [19]. At 25 °C, the homologous temperature is below 0.5; hence, the risk for creep deformation is low. Creep deformation should be monitored at 85 °C as the homologous temperature is 0.52.

TLP bonding shows a small but statistically significant deviation from the nominal value during the drift measurement at 10 bars. The mean long-term drift is 0.05% FSO/h, which is 0.04 bars in 8 h. Depending on the application, this can be ignored. The SEM image shows a good bond with few voids, and the EDX analysis indicates the formation of an intermetallic phase. Further work could be carried out to repeat the tests with reduced pressure. Another possibility for optimization is to vary the thickness of the Sn/Ag coating for the TLP bond. This could lead to different alloys and further improve the long-term stability of the pressure sensor. The measurement results from the characteristic curve are the best in this work; further optimization is not necessary.

Sensors with reactive joined membranes showed the biggest drift at 10 bars, while the measurement results of the characteristic curve were strongly fluctuating. The SEM images indicate a good joint. The reason for the drift is most probably the use of tin solder and its tendency for creep deformation. Reactive joining is possible with different material combinations. In this work, tin solder with nickel–aluminum RF was used. Further research could be carried out with high-melting solder to improve the sensor performance. This also requires the use of a different RF foil to generate more heat to melt the solder. A possible damage of the PCB material due to increased thermal stress must be monitored.

Sensors produced by welding were among the best results regarding drift at 10 bars. Nevertheless, these sensors suffer from a strong hysteresis in the characteristic curve. The voids, which can be seen in the SEM images, could be a reason. Further work should optimize this issue by variation of the process parameters, the layout of the welding spots, or the PCB material.

Sensors with sintered membranes offer good performance with regard to all investigated properties. The characteristic curve is not quite as good as for sensors with TLP bonding, but the average error is well below 1% FSO. The SEM images indicate lots of pores; process optimization to reduce them could lead to improved characteristic curves. No drift was measured.

**Proof** **of** **Theorem** **1.** The hypothesis, that drift effects are related to the joining method of the membrane and the PCB, can be confirmed. Other effects are unlikely, as sintering and welding do not show any statistically relevant long-term drift. The correlation between creep deformation and the calculated homologous temperature of the joining materials can be confirmed as well. For silver, which was used for sintering, the calculated temperature for creep deformation is above the test temperature, and no statistically significant drift was measured. □

To compare the measurement results with commercial pressure sensors, a quick market research was conducted. Similar sensors regarding measurement range, size, housing type, electrical interface, and cost are compared with the sensor of this work in Table 4.

Sintering, welding, and TLP bonding are furthermore suitable for higher operating temperatures than 85 °C. Reactive joining can be adapted for higher temperatures by changing the solder alloy and the RF. Combined with high-temperature PCB materials, such as PTFE, PI, or ceramic circuit boards, and high-temperature electronics, such as SOI CMOS high-temperature electronic circuits, a media-resistant pressure sensor with a temperature range of up to 200–250 °C is possible. Further research could be carried out in this area.

In this work, SEM images were used to analyze cross sections for pores or voids of the joints. In further work, nondestructive methods, e.g., scanning acoustic microscope (SAM) images or computed tomography scans (CT) could be used in addition. Initial studies on this have already been performed; an SAM image is included in the supplementary material in Figure S5. Another refinement of the analysis methods could be the use of transmission electron microscopy (TEM) to determine the exact alloys in TLP bonds instead of using energy-dispersive X-ray spectroscopy (EDX) to map the elemental distribution.

## 5. Conclusions

This investigation compares four methods, reactive joining, TPL bonding, sintering, and welding, to join the membrane on the PCB for the presented pressure sensor. Their essential quality features are investigated and compared with each other, namely measurement range, operating temperature, and long-term stability in a measurement range up to 10 bars.

The SEM images indicate good properties of the joints except for welding, where numerous cracks are visible and further optimizations of the process parameters are necessary. The EDX elemental analysis for TLP bonding is particularly interesting, as formations of intermetallic phases are indicated due to the elemental distribution.

The measurement of the long-term drift at room temperature shows a good correlation to the homologous temperature of the joining materials. A homologous temperature below 0.5 was calculated for sintering and TLP bonding, providing a theoretical reference for no-drift condition, which was confirmed by the associated measurements, showing low or no long-term drifts. The tin solder used for reactive joining has a homologous temperature above 0.5, and a long-term drift was measured as expected.

The measurements of the characteristic curves showed the best results for TLP bonding and sintering. Electric resistance welding offers best properties for high-volume production, as material costs and process times are low. For low-volume production, silver sintering is a good alternative, as process equipment cost is low.

Overall, it can be concluded that the presented pressure sensor can be used at up to 10 bars, as it offers acceptable measurement results for the investigated accuracy-related factors.

## Figures and Tables

**Table 2:** Methods used in this work to connect the membrane with the PCB.

	Electric Resistance Welding	Reactive Joining	Silver Sintering	TLP Bonding
Duration of the process preparation ^1^	<1 min.	<1 min.	<1 min.	~2.5 min.
Process time	<1 min.	<1 min.	120 min.	30 min.
Required process energy	Medium	Low	High	High
Process material cost	Low	High	High	High
Process equipment cost	High	Low	Low	High

^1^ Includes the handling of the membrane and the PCB and the preparation of the machine or apparatus.

**Table 3:** Homologous temperature for the used materials.

	SAC305 Solder	Sn	Nanoscale Silver Particles	Cu6Sn5	1.4435	CuSn8
T_M_	217 °C [31]	232 °C [32]	900 °C [18]	610 °C ^1^	1410 °C [33]	1040 °C [34]
T_H_, T = 25 °C	0.61	0.59	0.25	0.41	0.18	0.23
T_H_, T = 85 °C	0.73	0.71	0.31	0.45	0.21	0.27

^1^ Read from the phase diagram for Cu–Sn [19].

**Table 4:** Comparison of technical data with commercial sensors.

	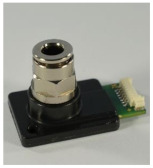 This work	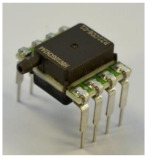 Amphenol ELVH-B010G-HRND-C-N2A4	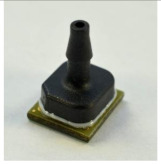 Honeywell ABP2LANT010 BG2A3XX
Measurement range	0–10 bars	0–10 bars	0–10 bars
Interface	SPI	I2C	I2C
Dimensions	23 × 39 × 26 mm^3^	10 × 13 × 6 mm^3^	8 × 7 × 11 mm^3^
Media resistant	Yes ^1^	Not specified	Liquid media capable
Accuracy	±0.4% FSO ^2,3^	±0.25% FSO ^4^	±0.25% FSO ^4^
Long-term stability	±0.1% FSO ^2,5^	Not specified	±0.25% FSO
Cost; purchase quantity 100 units	Target < EUR 10/unit	EUR 10.55/unit ^6^ [36]	EUR 21.22/unit ^6^ [37]

^1^ 1.4435 stainless-steel membrane or CuSn8 membrane with polymer housing. ^2^ Sensors with TLP-bonded membrane. ^3^ Maximum measured error including all errors due to pressure nonlinearity, pressure hysteresis, and nonrepeatability at room temperature. ^4^ The maximum deviation in output from a best fit straight line (BFSL) fitted to the output measured over the pressure range at 25 °C. Includes all errors due to pressure nonlinearity, pressure hysteresis, and nonrepeatability. ^5^ Measured at maximum pressure for 8 h. ^6^ Price research was conducted at the distributors Mouser Electronics, Digi-Key Electronics, Farnell, and RS Components. The lowest price with simultaneous availability was included in the table.

## Data Availability

All data used are shown in the text and the supplementary material. Raw data are available on request.

## Supplementary Materials

The following are available online at https://www.mdpi.com/article/10.3390/s21165557/s1, Figure S1: FEM simulation model, Figure S2: FEM simulation model; Figure S3: Simulation result; Figure S4: Relaxation test; Table S1: Generically determined creep parameters for SnAgCu solders; Table S2: Material data; Equation S1: Garofalo’s formula for creep deformation; Figure S5: SAM image of a reactive joined Membrane.
