# Supplementary material for: Characterization of a PCB Based Pressure Sensor and Its Joining Methods for the Metal Membrane
Source: Sensors (Basel). 2021 Aug 18;21(16):5557. doi: 10.3390/s21165557 (PMC8402279; doi:10.3390/s21165557)
Supplement: Supplementary file 1 [file sensors-21-05557-s001.zip › sensors-1331501-supplementary.pdf]

sensors

MDPI

Supplementary Materials

PCB Based Pressure Sensor with Metal Membrane

$$\frac{d\varepsilon_{cr}}{dt} = C * [sinh(\alpha * \sigma)]^n * e^{\left(-\frac{Q_a}{k*T}\right)} \qquad [28] \qquad \text{Equation S1}$$

Table S1. Generically determined creep parameters for SnAgCu solder [28].

Sn95.5Ag3.8Cu0.7<br>Sn95.75Ag3.5Cu0.75<br>Sn96.5Ag3Cu0.5	C[s^{-1}]	277984
	A[MPa^{-1}]	0,02447
	n	6.41
	Q_a[eV]	0.56

Table S2. Material data [29].

Part	Material	E-Module [MPa]	Tensile Strength [Mpa]	Fatique Limit [Mpa]	Material Laws
Membrane	CuSn8	92.800	749	225	Linear-elastic
Solder	SAC305	50.000	-	-	Creep deformation
Solder pad	Cu	110.000	430		Linear-elastic
PCB	FR4	19.300			

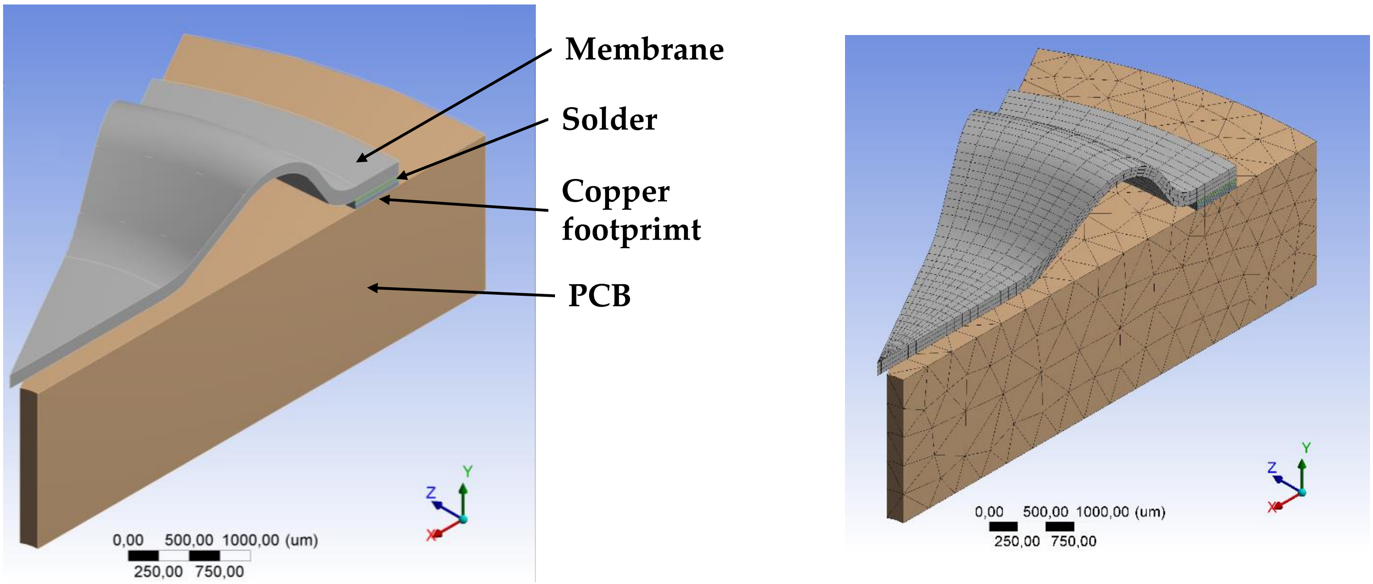

(**a**) (**b**)

Figure S1. FEM simulation model; (**a**) A rotational 1/12 model was simulated; (**b**) Mesh.

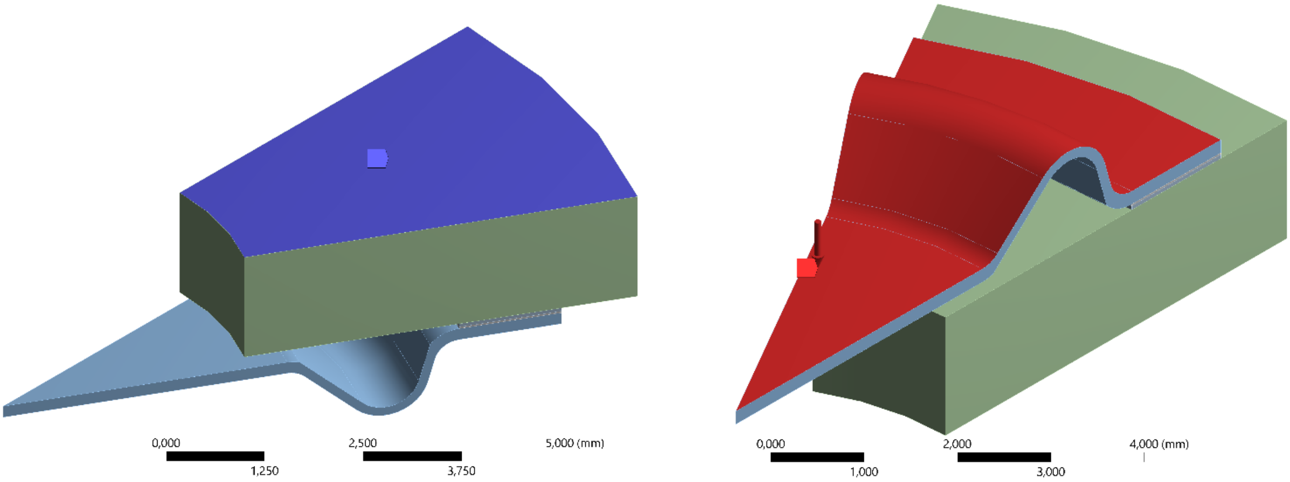

(**a**) (**b**)

Figure S2. FEM simulation model; (**a**) Fixed bearing at the bottom of the PCB; (**b**) Pressure is ap-plied on the surface of the membrane.

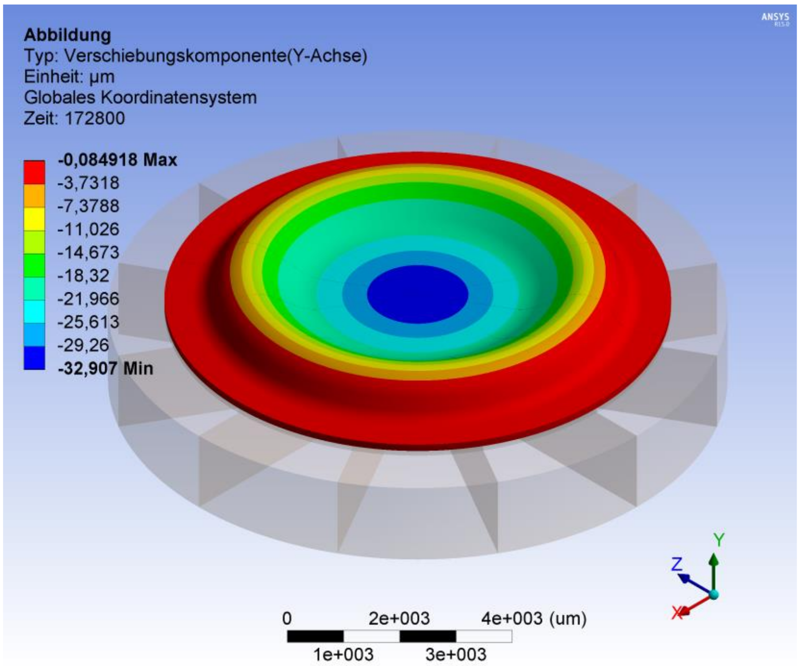

Figure S3. Simulation result: Displacement component in Y-direction.

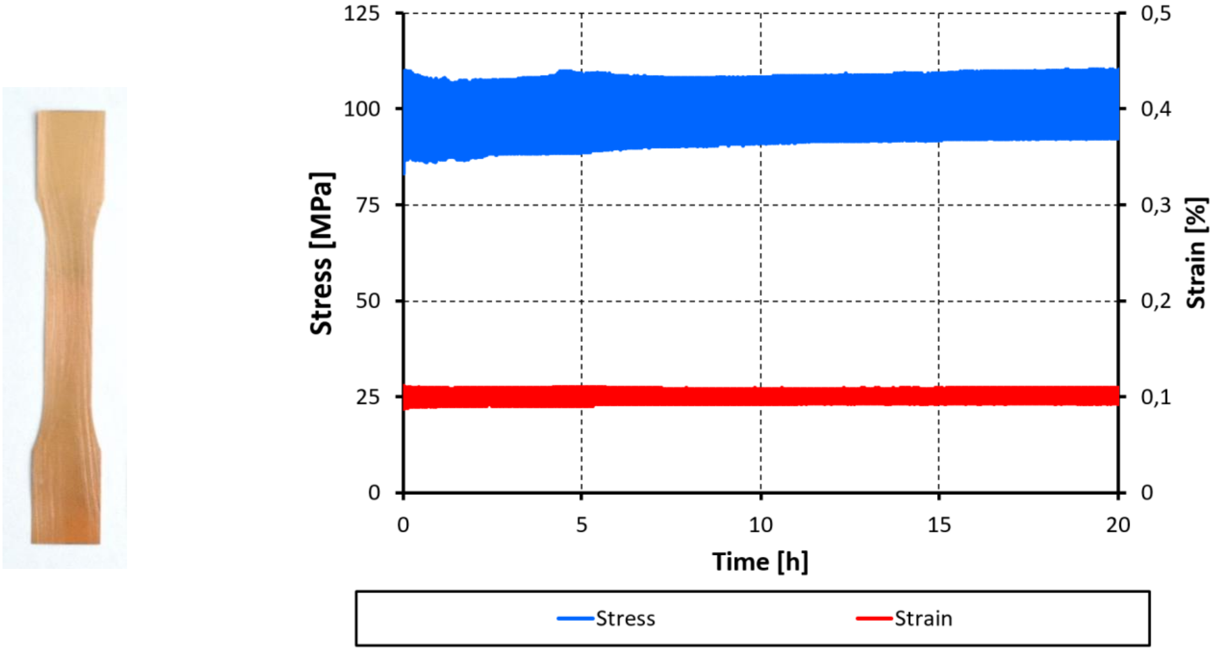

(**a**) (**b**)

Figure S4. Relaxation test: (**a**) Test sample; sample length: 115 mm, sample with: 12.5 mm, sample thickness: 50µm, strain: 0.1%; (**b**) Measurement result; Measuring instrument: TiraTest 2810 with environmental chamber, temperature: 125° C.

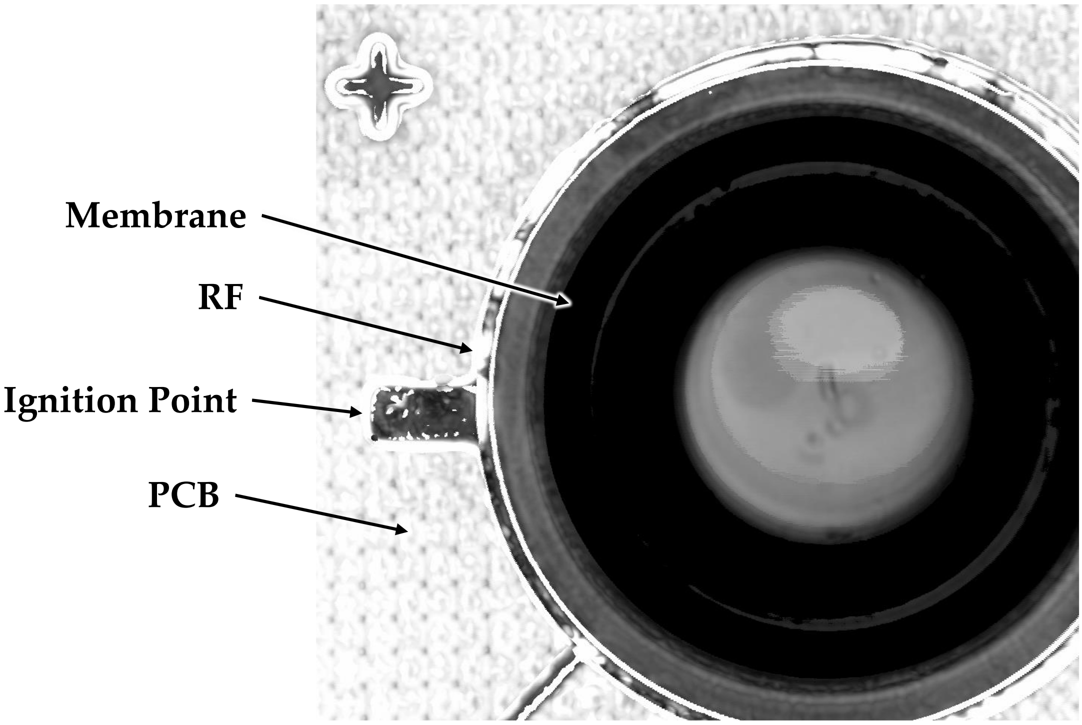

Figure S5. SAM image of a reactive joined membrane.
